# The Interaction between Feed Bioactive Compounds and Chicken Genome

**DOI:** 10.3390/ani13111831

**Published:** 2023-05-31

**Authors:** Kristina Gvozdanović, Zlata Kralik, Žarko Radišić, Manuela Košević, Gordana Kralik, Ivona Djurkin Kušec

**Affiliations:** 1 Faculty of Agrobiotechnical Sciences Osijek, Josip Juraj Strossmayer University of Osijek, Vladimira Preloga 1, 31000 Osijek, Croatia; 2 Scientific Center of Excellence for Personalized Health Care, Josip Juraj Strossmayer University of Osijek, Trg Svetog Trojstva 3, 31000 Osijek, Croatia; 3 Nutricin j.d.o.o., Đure Đakovića 6, 31326 Darda, Croatia

**Keywords:** chicken, gene expression, nutrigenomics, animal feed

## Abstract

**Simple Summary:**

The addition of various bioactive compounds to chicken feed has a positive effect on the health, production traits and general welfare of the animals. To use these compounds more efficiently, it is necessary to better understand the molecular relationship between the nutrients in the feed and the genes that trigger the change to a preferred phenotype. This can be achieved with the help of nutrigenomics, a science that studies the interaction between nutrients and the genome and their influence on metabolic and physiological processes in the body. By implementing the knowledge gained from nutrigenomics, it is possible to more efficiently produce high-quality chicken products that have a positive impact on human health.

**Abstract:**

Consumer demand for high quality and safe foods that will have a positive impact on their health has increased in recent years. Today, it is possible to meet those demands by combining the genetic potential of domestic animals and applying different feeding strategies. Nutrigenomics is one of the “omics” sciences that studies the interaction between nutrients and the genome together with their influence on metabolic and physiological processes in the body. While nutrition of domestic animals is solely based on studying the influence of nutrients on animal health and production traits, nutrigenomics integrates the fields of nutrition, genomics, molecular genetics and bioinformatics. By understanding the molecular relationships between different forms and/or concentrations of nutrients in feed and genes, it is possible to answer the question of how small changes in the diet of farm animals can produce a quality product with positive effects on human health. The aim of this article is to describe how the manipulation of adding different nutrients in the feed affects the expression of different genes in chicken and consequently alters their phenotype.

## 1. Introduction

**Figure 1:**
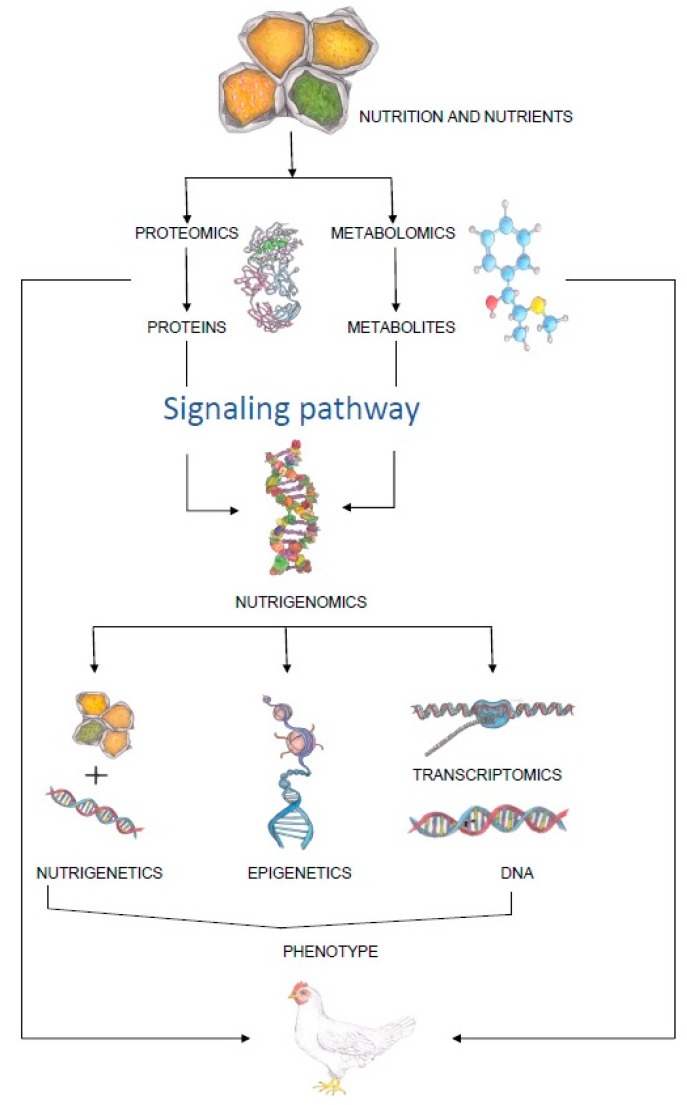
Schematic representation of the influence of feed on the phenotype of animals.

The importance of consuming healthy food of good quality has been known for almost two thousand years. Terms, such as “food” and “nutrition”, have been known and used since the earliest history, although the concept of nutritional science as we know it today emerged in the early 19th century [1]. The concept reached its peak after the isolation of vitamin C in 1936, when it was officially documented for the first time that this vitamin protects against scurvy [2]. Thereafter, nutrition science has continued to evolve, from the discovery of the role of fat and sugar in the body in the 1950s, through dietary supplements and nutrition adjusted to chronic diseases in the 1970s, to a science-based personalized approach to individual nutrition in the 21st century [2]. However, none of this would have been possible without the development of technology and different sciences, and in particular “omics” sciences. In the context of nutrition, this refers predominantly to nutrigenomics, which is defined as a scientific discipline that studies the relationships between nutrients, diet and gene expression [3]. The aim of nutrigenomics is to find out how food components (bioactive components) can influence gene expression in terms of enhancing or suppressing their potential [4]. With its tools, it is possible to select very precisely the nutrients that can fine-tune the expression of genes in humans or animals and improve their health or production traits [5]. Before the advent of nutrigenomics in livestock production, nutrition and genetics were studied as separate disciplines, without taking into consideration the influence of genome–nutrition interaction effect on metabolic and physiological processes in domestic animals [6]. However, with the development of epigenetics, genomics and other “omics” sciences, it became clear that the expression of certain traits in domestic animals is influenced by the synergy of genetic background and environmental factors [7]. An illustration of how “omics” science is involved in the expression of a particular phenotype can be found in Figure 1.

Food components interact with the body at system, organ, cellular and molecular levels. They are present in complex mixtures, in which the presence and concentrations of a single compound and also the interactions of several compounds, determine their bioavailability and biological efficiency.

The interaction between nutrients and cellular/genetic processes is referred to as nutrigenomics. It is a broad term that encompasses nutrigenetics, which aims to understand how genetic background affects the body’s response to bioactive food ingredients; epigenetics, which studies stable, environmentally induced changes in gene expression; and transcriptomics, which studies all forms of RNA in the body. Proteomics in nutrition can identify and quantify bioactive proteins and peptides and address questions of nutritional biological efficiency, while metabolomics aims to determine metabolites responsible for a particular phenotype. In this way, the coherent measures of nutrigenomics, proteomics and metabolomics play a crucial role in understanding how a particular diet interacts with the body and forms a particular phenotype.

A better understanding of the physiology of domestic animals and its relationship to the nutrients in diets is a prerequisite for designing meals that not only have a positive impact on production results but also enable the realization of the animal’s full genetic potential [8]. Recent research has shown that components, such as vitamin E [9], carotenoids [10], coenzyme Q [11], organic acids [12], essential oils [13], amino acids [14] and many other bioactive compounds, affect gene expression in different species of domestic animals, confirming the existence of interactions between nutrients in the diets and their genome. With the production of 100.974 million tons of meat and 86.670 million tons of eggs in 2020, poultry breeding is the leading livestock industry in the world [15].

From the consumer’s perspective, poultry meat and products are perceived as low-fat and lean foods that are healthier than other products derived from farmed animals. This is one of the reasons why consumption of poultry meat has increased enormously and poultry meat ranks first in global meat consumption. It also has the organoleptic and qualitative characteristics desired by consumers, such as a neutral taste, a uniform and good texture and a light color [15]. From a nutritional point of view, it has a high protein content, a low cholesterol and fat content and a balanced ratio of n-6 to n-3 polyunsaturated fatty acids.

Feed is the most important element in the production system, the cost of which can account for up to 70% of total production costs. The importance of feed optimization in poultry nutrition is reflected in the optimal distribution and utilization of nutrients, which leads to improved performance of the animals and furthermore to a minimization of economic losses and an increase in profitability [16,17]. Due to the increasing consumption of poultry products, feed optimization represents one of the most important tasks of the farm, which would neither be possible nor sustainable without the application of findings from nutrigenomics.

The aim of this paper is to summarize new scientific findings on the impact of feed modulation using bioactive substances on health and productive traits of chicken.

Although there are many bioactive compounds that have been shown to have a positive effect on the health and productive traits or on the expression of various genes in chicken, only scientific research that have been shown to act on both the phenome and the genome are presented in this article.

## 2. Phytonutrients

Phytochemicals are substances of plant origin whose addition to animal feed positively influences growth, production traits and maintenance of intestinal microflora [18,19]. The results of numerous studies have shown that essential oils and substances can play an important role in poultry health and performance by stimulating food intake, secretion of endogenous enzymes, production of antioxidants and antibacterial activity. This group includes plant extracts and their active ingredients, such as carvacrol, thymol and capsaicin, with beneficial effects related to their bioactive compounds [20]. Phytonutrients can be added to poultry feed individually or as mixtures in varying ratios. Phytonutrients, such as oregano and cinnamaldehyde, are considered natural additives that help protect broilers from infectious diseases [21] while providing an alternative to antibiotics as growth promoters [22]. Furthermore, it has been shown that the addition of oregano and cinnamaldehyde to broiler diets stimulates the expression of the insulin-like growth factor 1 (*IGF1*) and glyceraldehyde-3-phosphate dehydrogenase (*GAPDH*) genes and positively affects production traits, such as carcass yield, gut microflora, and also blood glucose and cholesterol levels [23]. Cinnamaldehyde also has a positive effect on interleukin (*IL-10*) and transforming growth factor-beta (*TGF-β*) gene expression in intraepithelial lymphocytes [8]. Numerous studies have shown that the addition of curcumin to poultry feed positively influences the expression of genes important for lipid and glycogen metabolism. For example, Xie et al. (2019) [24] showed that the addition of curcumin to a broiler diet significantly reduced the expression of genes for acetyl-CoA carboxylase (*ACC*), apoptosis antigen 1 (*FAS* receptor) and transcription factor *SREBP-1c*, all of which are involved in lipogenesis and fatty acid synthesis in poultry, playing an important role in reducing abdominal fat deposition. Hafez et al. (2022) [25] investigated the effect of curcumin on the expression of genes related to poultry growth. The authors showed that the addition of curcumin had a positive effect on poultry growth by increasing the expression of insulin-like growth factor 1 (*IGF-1)* and growth hormone receptor (*GHR*) and decreasing the expression of leptin (*LEP*) and myostatin (*MSTN*). McKnight et al. (2019) [26] analyzed the effect of the addition of fatty acids, organic acids and phytochemicals on the morphology and expression of inflammatory genes in broilers. The results showed microstructural changes in the duodenum and jejunum of broilers that consumed feed with added bacitracin methylene disalicylate (BMD) and fatty acids as well as higher expression of the cytokines interferon Ɣ (*IFNƔ*), interleukin 6 (*IL-6*) and inducible nitric oxide synthase (*iNOS*). Previous studies confirmed that *IFNƔ* has an inhibitory effect on parasitic diseases and is considered a key component of the immune response to parasitic infections in broilers [27,28].

Neem leaf extract (*Azadirachta indica*) is another phytochemical with a proven positive effect on broiler meat quality and also on the gene expression of antioxidant enzymes in the breast muscle. It should be, however, emphasized that fresh neem leaves can negatively affect broiler growth due to their high crude fiber content [29], because of which it is necessary to add them in the form of a dry leaf extract. The results of Nakamura et al. (2022) [30] showed that the addition of 2.0% neem dry extract to broiler feed increased the expression of genes important for the activity of antioxidant enzymes in the breast muscle (Cu/Zn superoxide dismutase—*SOD1*, Mn superoxidase—*MnSOD*, glutathione peroxidase 7—*GPX* 7 and catalase—*CAT*). Furthermore, the authors showed that the addition of the neem to broiler feed reduces lipid peroxidation and water losses in *m. pectoralis major* muscle.

In addition to micronutrients, the addition of plant by-products to feed can also influence gene expression in poultry. One such by-product is the water produced during the extraction of olive oil. Sabino et al. (2018) [31] showed a positive effect of adding this by-product to broiler feed on their small intestinal function and health in general. The authors reported differential expression of 280 genes related to lipid metabolism and the regulation of virus replication in the epithelial cells of the jejunum of broilers fed experimental diets.

## 3. Vitamins and Minerals

Vitamin A (retinol) has an important function in both avian embryonic development and adult biology [32]. It is a fat-soluble vitamin that can be stored efficiently in liver and egg yolk. However, its amounts should be carefully managed, as excessive amounts of this vitamin remaining in the bird’s body can lead to vitamin D deficiency [33], reduce the deposition of α-tocopherol in the yolk [34] or even be toxic to the brain and liver [35]. Vitamin A can be easily destroyed during feed processing, so it needs to be supplemented. The study by Yuan et al. (2014) [35] showed that supplementation of vitamin A at a dose of up to 35,000 IU/kg did not affect the reproductive performance of the birds and increased the vitamin A concentration in liver and yolk, but decreased the α-, γ- and total tocopherol concentration in yolk and the α-tocopherol in liver. In contrast, at a dose of 45,000 IU/kg and above, egg weight, yolk color, eggshell thickness and firmness, and reproductive performance decreased significantly. The authors reported that the addition of vitamin A to the diet increased mRNA expression of the vitamin D receptor in the duodenal mucosa, increased aspartate aminotransferase activity and decreased serum total bilirubin concentration.

Vitamin D3 plays an important role in maintaining phosphorus homeostasis, enhancing intestinal absorption of phosphorus and stimulating osteoblast activation and proliferation. Vitamin D requirements depend on dietary Ca and P concentrations and are estimated to be higher than recommended levels in the first two weeks of a broiler’s life [36]. The study by Shao et al. (2019) [37] showed that vitamin D3 supplementation in broiler diets promoted intestinal P absorption and bone P utilization, and concluded that this effect may be related to increased *PiT-1* levels in the duodenum and *PiT-1* and *NaP-IIb* levels in the jejunum, respectively. In addition, vitamin D supplementation was shown to have an immunomodulatory effect in chickens. Supplementation of Ca- and P-deficient diet with vitamin D increased transcription of *TLR2b*, *TLR4*, *CATH1* and *CATHB1* and predominantly *Th2* cytokines in the spleen, while supplementation of the control diet with vitamin D downregulated *TLR4* transcription and increased *CATH1*, *CATHB1*, *Th1* and *Th2* cytokine transcription in a dose-dependent manner [36].

Apart from being the most important vitamin related to blood coagulation, vitamin K, together with vitamin D, plays a crucial role in bone turnover and strength and also has an anti-inflammatory effect in the body [38]. The addition of vitamin K3 and probiotics has been shown to promote growth performance of broiler chickens in grower phase by synergistically improving the physical and chemical properties of the tibia through modulation of calcium and phosphorus metabolism and expression of osteogenic genes (runt-related transcription factor 2 *OCN,* and alkaline phosphatase) [39].

Calcium is considered one of the most important minerals in animal diet due to its crucial role in bone development and growth. Although it is generally always added to feed in its inorganic form, it is necessary to maintain calcium’s balance in the body as its deficiency can lead to bone injury and poor growth [40], and its presence in excessive amount can also reduce body weight gain and feed intake of broilers [41]. The response of the animal body through growth and bone quality to dietary calcium depends on calcium absorption in the small intestine. Han et al. (2022) [42] found that low dietary Ca stimulates transcription of the *VDR* (nuclear vitamin D receptor (*nVDR*) and membrane vitamin D receptor (*mVDR*)) and the combination of vitamin D with *VDR* to regulate Ca absorption in the small intestine, while high Ca inhibits this effect and prevents excessive absorption in the small intestine of broiler chickens.

Vitamin C is a water-soluble antioxidant compound whose main role is to protect cells from oxidative damage and to strengthen the immune system [43]. It is not a part of any metabolic pathway but acts as an essential cofactor in many enzymatic reactions, such as the synthesis of collagen, carnitine and catecholamine and the metabolism of microsomes or the synthesis and catabolism of tyrosine [44]. Due to its positive effect on maintaining the integrity of the cellular defense system during stress, vitamin C administered together with vitamin E may adverse the performance deterioration of chickens that occurs during heat stress [45]. Furthermore, a study by Shakeri et al. (2020) [44] showed that in broiler chickens exposed to summer heat stress, the addition of 200 mg/kg vitamin C and 100 mg/kg vitamin E to the basal diet significantly decreased the expression of interleukin *IL-1β*, *IL-*6, interferon *(IFN)-*γ, toll-like receptor (*TLR)-*4 and *HSP70* in the liver, all of which are associated with response to stress. Similarly, Abdel-Moneim et al. (2021) [46] found that the addition of 200 mg/kg vitamin C alone provided protection for broiler chickens against the risk of high density through improved total feed intake, reduced mortality and down-regulation of *HSP70* expression levels in the liver.

Vitamin E and selenium are bioactive substances with a positive influence on chicken health, production traits and the quality of their products [47,48,49]. Selenium is an essential element found in the composition of glutathione peroxidase (GPx), an enzyme important in lipid peroxidation processes, while vitamin E plays an important role in protecting cells from free radicals [48]. The recommended levels of selenium and vitamin E in broiler feed are 0.15 mg/kg and 10 IU vitamin E/kg, respectively [50]. Khalifa et al. (2021) [51] investigated the synergistic effect of selenium and vitamin E addition on the growth and expression of genes related to growth in broilers. The authors showed that selenium and vitamin E influence broiler growth by regulating *IGF-1* and *GHR* genes. According to Kirella et al. (2021) [52], the expression of genes related to poultry growth can be increased by adding by-products of agricultural production with high concentrations of vitamin E to feed. On the other hand, the addition of vitamin E isomers, α-tocopherol and γ-tocopherol, affect the expression of genes related to lipid metabolism and genes responsible for inflammatory processes and the immune response [53]. Shehata et al. (2022) [54] reported that the addition of vitamin E in feed reduced the negative effects of poor housing conditions (e.g., high stocking density) on growth traits and stress in broiler chickens. The results of their study showed increased expression of the genes *GHR* and *IGF-1* and a significant decrease in triiodothyronine (T3) and thyroxine (T4), circulating thyroid hormones known to be susceptible to stressful conditions [55]. According to Khalifa et al. (2021) [51], the antioxidant effects of vitamin E and selenium were also reflected in the good health of broiler chickens. The findings of Khalifa et al. (2021) [51] were confirmed by Elgendey et al. (2022) [56], who also reported an increase in *CAT* and *SOD* expression. *CAT* and *SOD* encode the levels of the antioxidant enzyme GPx. In addition, Amevor et al. (2021) [57] reported a synergistic effect of vitamin E and quercetin, due to which higher expressions of interferon γ (*INF-γ*) and interleukin 2 (*IL-2*), which are important for the immune system, were found in the liver of broilers who were fed these compounds. 

The addition of selenium and vitamin E to chicken feed affects also the expression of genes involved in the transport of nutrients in the gut [58], genes expressed in the fallopian tube tissue [59], genes related to the occurrence of oxidative stress, and those related to inflammation-related disorders [56]. The combination of n-3 PUFA fatty acids and vitamin E increases the expression of genes related to peptide transport (*SLC15A1*, and *GALNT2*), oxidative stress and intestinal hypoxia, as well as some genes related to the occurrence of stress (mucin 2-*MUC2*, interferon gamma-*IFNG*, member 7 of the heat stress protein group-*HSPB7* [58]. Furthermore, adding selenium and vitamin E to chicken feed affects the expression of genes involved in the transport of nutrients in the intestine [58], genes expressed in oviductal tissue [59], genes related to the occurrence of oxidative stress and genes related to inflammation-related disorders [56]. Because of their rapid growth and ability to store a large amount of abdominal fat, broiler chickens are a good model for studying carcass lipid and abdominal obesity [60]. In this context, research by Zhang et al. (2021) [9] has shown that vitamin E supplementation in broiler feed reduces the expression of genes leading to de novo synthesis of fatty acids (*FASN*, *ACACA*), thereby reducing the accumulation of abdominal adipose tissue.

## 4. Flavonoids and Carotenoids

Poultry, especially those used for the production of meat (broilers) and eggs (hens), due to extremely strong selection pressure on high productivity, are very sensitive to oxidative stress—a condition in which more reactive oxygen compounds (ROS) and reactive nitrogen compounds (RNS) are present than can be broken down by the animal’s organism. Furthermore, commercial production of broilers and laying hens is associated with numerous environmental stressors (vaccination, density in the facility, long photoperiods, dust, and ammonia) that predispose them to oxidative stress [61]. Previous research has shown that oxidative stress has a tremendous impact on poultry health and performance [62]. For this reason, flavonoids, in the form of leaves or fruits of various plants, are being increasingly added to animal feed in modern poultry production. Flavonoids are polyphenolic secondary metabolites of plants found in a variety of foods, including berries, grapes, onions and legumes [63,64]. They are synthesized from the amino acids phenylalanine and malonate. To date, more than 6000 different flavonoid compounds with influence on animal growth, reproduction and the immune system are recognized [65].

Ouyang et al. (2016) [66] investigated the effect of flavonoids from alfalfa (*Medicago sativa*) on the expression of genes related to lipid metabolism in the liver and adipose tissue of broiler chickens. The addition of alfalfa flavonoids to diets at 5, 10 and 15 mg kg^−1^ lowered the expression of the fatty acid synthase gene (*FAS*), which catalyzed the final step of the fatty acid synthesis, and increased the expression of lipoprotein lipase (*LPL*), peroxisome proliferator-activated receptor γ (*PPARγ*) and the adipose triglyceride lipase (*ATGL*). This suggests that supplementation of flavonoids from alfalfa have better antioxidant activity by regulating the activity of *LPL*, *PPARy* and *ATGL* genes compared to broiler chicken fed with basal diet.

The reproductive capacity of a laying hen decreases with age, and this is related to the weakening of the liver–blood–ovary system function together with reduced estrogen production. Research by Dai et al. (2021) [67] has shown that the addition of flavonoids from hawthorn to the diet of laying hens increases estrogen levels and slows ovarian apoptosis, by increasing the expression of the proliferating cell nuclear antigen (*PCNA*) gene. Studies also found increased expression of the *Nrf2* gene in the ovaries, as well as the apolipoprotein genes *ApoB* and *ApoV1*. The *Nrf2* gene is responsible for antioxidant activity and protection against oxidative stress in the ovaries of laying hens, while *ApoB* and *ApoV1* are important protein components of low-density lipoproteins involved in the maintenance of lipid homeostasis in the liver. Similarly, Shi et al. (2022) [68] reported that flavonoids from wormwood increased the expression of the *Nrf2* gene, which then translocated to the nucleus and bound to antioxidant elements. The authors concluded that flavonoids from wormwood could stimulate the expression of antioxidant enzymes and increase the activity of the antioxidant enzymes CAT, SOD and GPx, thus protecting the organism from oxidative stress. In addition to protecting the body from oxidative stress, flavonoids have also been shown to have an effect against osteoporosis. For example, Huang et al. (2020) [69] reported that the addition of total flavonoids from *Drynariae* rhizomes to the feed of laying hens influenced bone health by regulating osteoclast activity. The authors showed that the addition of 0.5 or 2.0 g/kg total flavonoids to the diets significantly increased the expression of the RUN transcription factor 2 (*RUNX2*) and osteoprotegerin (*OPG*) genes (responsible for osteoblast differentiation and bone mineral density), and decreased the expression of the receptor activator of nuclear factor kappa-Β ligand (*RANKL*) gene, associated with osteoclast activity.

Carotenoids are among the most widely distributed fat-soluble pigments found in various plants, microalgae, bacteria and fungi. Depending on their function, they are divided into two groups: carotenes (including lycopene, α-carotene and β-carotene) and xanthophylls (such as lutein and zeaxanthin) [70]. The animal and bird body cannot synthesize them by themselves, so they must be ingested. The role of carotenoids in improving the quality of meat and eggs in poultry production has been known for a long time [71,72], however, these pigments have recently received a lot of attention due to their bioactive properties and beneficial effects on animal health [73,74]. It has been found that carotenoids reduce oxidative stress in the host body through multiple cellular mechanisms (such as scavenging free radicals and upregulating the production of antioxidant enzymes) and thus have anti-cancer, immunomodulatory, anti-inflammatory, antibacterial, neuroprotective and anti-diabetic functions [75]. Csernus et al. (2020) [10] and Gao et al. (2012) [76] investigated the effect of carotenoid supplementation on the expression of the anti-inflammatory genes interleukin 1β (*IL-1β*), interleukin 6 (*IL-6*), interferon-α (*IFN-α*) and interferon-γ (*IFN-γ*) in poultry. Their results showed that carotenoids reduced the gene expression of inflammatory factors in stressed animals. Increased expression of anti-inflammatory cytokine genes leads to pathological states of the body’s immune system, and therefore it is important to maintain a balance between inflammatory and anti-inflammatory factors. The results of Gao et al. (2012) [76] showed that the addition of xanthophyll can reduce the expression of anti-inflammatory cytokines in the liver, duodenum and jejunum of chicken, thus helping to maintain the balance between inflammatory and anti-inflammatory factors.

## 5. Amino Acids

The addition of amino acids to feed has several positive effects on chicken production traits and overall health: amino acids improve feed conversion, influence growth, have a positive effect on the immune system, reduce the effects of thermal stress to which chicken are exposed and have a positive effect on the development of muscle tissue [77]. At the cellular level, their function is extremely important as they are substrates for protein synthesis and are involved in the control of gene expression through their ability to modulate the initiation phase of mRNA translation in poultry [78]. Amino acid signaling occurs through two pathways: the mechanistic target of rapamycin complex 1 (mTORC1) and the amino acid response pathway (AAR) [79]. Oral intake of nutrients, such as amino acids, induces transcription, RNA stability and processing, protein synthesis and modification. These processes influence DNA replication and the regulation of gene expression in mammalian and avian cells. Therefore, the addition or absence of exogenous amino acids can effectively regulate gene expression in mammals and birds [79].

Among all amino acids, lysine is considered to be essential and for limiting the growth of the animal. This amino acid is important for the synthesis of proteins, cytokines, gene expression and the response of the immune system to infections [79]. Analysis of mRNA expression from broiler chickens fed with low and high levels of lysine identified increased expression of 67 genes and reduced expression of 143 genes related to cell growth, respectively. Methionine is another amino acid whose deficiency affects mRNA expression and thus growth and immune function in poultry [80,81]. For example, it has been observed that broilers that grow more slowly consume larger amounts of feed containing non-essential amino acids (alanine, asparagine, and aspartic acid), while broilers that grow quickly consume more feed with the addition of essential amino acids (methionine, lysine, and threonine) [82].

In vertebrates, histidine-bound dipeptides are found in relevant concentrations in skeletal muscle, cardiac muscle and some parts of the brain. These dipeptides can act as intracellular buffers, metal ion chelators or antioxidants. In chickens, the dominant dipeptide related to histidine is called carnosine and it consists of histidine, the non-essential amino acid β-alanine and the methylated form of anserine [83]. The secondary precursor of carnosine, β-alanine, is formed as an intermediate in the metabolic pathways of aspartate and uracil [83]. Histidine deficiency leads to a decrease in body weight and an imbalance of body nitrogen [84], while reduced carnosine concentration has been found in birds suffering from pectoral myopathy [85]. Therefore, it is hypothesized that an increased intake of histidine may help to increase carnosine concentrations and thus prevent the development of pectoral myopathy [83]. However, it is important to note that the highest carnosine levels are synthesized by the addition of β-alanine and L-histidine combination, and that increased carnosine content does not negatively affect the quality of the meat, but rather improves the texture of the meat and alters the secondary protein structures [86]. In addition, increasing carnosine content in meat improves the oxidative stability of meat [87], reduces drip loss, cooking loss and tenderness of the meat [88], and also increases the initial and final pH values of *M. pectoralis* in chicken [89]. On the cellular level, supplementation with β-alanine resulted in multiple increases in carnosine synthase (*CARNS1*) and taurine transporter (*SLC6A6*; [89]). The same group of authors found that individual addition of β-alanine and L-histidine to broiler meal increased the expression of the histidine carboxylase, carnosine synthase, peptide transporter 1 (*PEPT1*) and inorganic phosphate 1 (*PHT1*) transporter genes, while the combination of L-histidine and β-alanine only increased the expression of the *PHT1* transporter gene. The *PEPT1* gene is a member of the *POT* family of membrane transporters that uses the proton electrochemical gradient to drive the uptake of di- and tripeptides across cell membranes [90], and is therefore capable of transporting both L-histidine and carnosine in skeletal muscle [14].

Kubota et al. (2021) [91] analyzed the RNA profile of Korat chicken supplemented with β-alanine and L-histidine, which resulted in a very tender breast muscle. The authors noted increased expression of genes involved in the regulation of myosin, intramuscular fat and calpain (*LOC107051274*, *ACSBG1*, and *CAPNS2*) and decreased expression of myosin VIIB (*MYO7B*), myosin binding protein H (*MYBPH*), inhibitor of serpin peptidase H (*SERPINH1*) and phosphoglycerate mutase 1 (*PGAM1*) genes. Interestingly, no carnosine synthase was detected. Functional enrichment analysis identified signaling pathways affected by dietary supplements, including the insulin signaling pathway (β-alanine supplementation) and insulin resistance, and adipocytokine signaling pathways (L-histidine supplementation).

L-carnitine is an amino acid with an irreplaceable role in intermediate metabolism. Its role in poultry production is multifunctional: it influences growth, strengthens the immune system, improves semen quality and has an antioxidant effect. Its concentration varies considerably depending on the species, tissue type and nutritional status of the animal [92]. The addition of L-carnitine to broiler water at a dose of 50 mg/kg/day during 35 days showed significant upregulation of amino acid cation transporter (*CAT2*), myoblast determination protein 1 (*MYOD*) and myogenic factor 5 (*MYF5*), the genes that play an important role in muscle growth and proliferation in animals and birds [93]. The results showed a significant increase in live weight and a decrease in feed intake in broilers that were supplemented with L-carnitine compared to the control group. Similarly, the introduction of a combination of L-carnitine and a methionine derivate, methyl methioninenine sulfonium chloride (MMSK), into broiler feed showed that the addition of MMSK alone or in combination with L-carnitine reduced the expression level of the *MSTN* gene and increased the expression of the *IGF-1* gene [94]. Higher growth rates and live weight were observed in broilers with increased expression of the *IGF-1* gene and reduced expression of the *MSTN* gene. The obtained results were expected, considering that the *IGF-1* gene promotes growth and development of the organism, while the *MSTN* gene is associated with muscle growth, i.e., inhibition of its expression leads to an increase in muscle mass.

## 6. Conclusions

The addition of various bioactive compounds to the feed improves the production traits, health and metabolism of chickens by altering the expression profile of genes involved in various metabolic pathways. The addition of phytonutrients and flavonoids positively influences the expression of genes important for lipid and glycogen metabolism, together with genes related to poultry growth and oxidative mechanisms, while modification of chicken feed with amino acids, vitamins or minerals influences signaling pathways responsible for the balance of inflammatory responses, improves production traits and early embryonic development.

Future research in the field of nutrigenomics will lead to the combined use of different technologies, the results of which will contribute to a better understanding of the interaction between nutrition and biological processes in the body and improve the possibility of combating disease through the effect of nutrients on the genome of farm animals. In addition, it will be possible to better control the intake of vitamins and minerals, preventing their excessive absorption and enabling the production of functional poultry products.

## Data Availability

Not applicable.
